# Amarogentin Inhibits Liver Cancer Cell Angiogenesis after Insufficient Radiofrequency Ablation via Affecting Stemness and the p53-Dependent VEGFA/Dll4/Notch1 Pathway

**DOI:** 10.1155/2020/5391058

**Published:** 2020-10-20

**Authors:** Yongchuan Zhang, Yinglin Zhang, Jin Wang, Haitao Gu

**Affiliations:** ^1^Department of Hepatopancreatobiliary Surgery, Third Hospital of Mianyang-Sichuan Mental Health Center, Mianyang, Sichuan 621000, China; ^2^Key Laboratory of Molecular Biology for Infectious Diseases of the Ministry of Education of China, Second Affiliated Hospital of Chongqing Medical University, Chongqing 400010, China; ^3^Department of Gastrointestinal Surgery, Second Affiliated Hospital of Chongqing Medical University, Chongqing 400010, China

## Abstract

**Background:**

Whether and how amarogentin suppresses the angiogenesis effect in liver cancer cells after insufficient radiofrequency ablation (iRFA) are still poorly studied.

**Methods:**

The number of liver cancer stem cells (LCSCs) and the level of vascular endothelial growth factor A (VEGFA) were assessed in liver cancer tissue after iRFA. Then, CD133-positive cells were detected in iRFA models of HepG2 and Huh7 cell lines treated with amarogentin. Tube formation assays were applied to observe the antiangiogenesis effects of amarogentin. In addition, the angiogenesis-related molecules p53, delta-like ligand 4 (Dll4), and Notch1 were detected in the iRFA cells and mouse models treated with amarogentin.

**Results:**

The mRNA and protein expression levels of CD133 and VEGFA were significantly higher in the residual liver cancer tissue than in the liver cancer tissues treated by hepatectomy. Amarogentin then markedly decreased the percentage of CD133-positive cells in the iRFA model in both HepG2 and Huh7 cell lines. The number of tubules formed by human umbilical vein endothelial cells (HUVECs) was significantly decreased by amarogentin. Inversely, the antiangiogenesis effect of amarogentin was counteracted after p53 silencing in the iRFA cell models.

**Conclusion:**

Amarogentin prevents the malignant transformation of liver cancer after iRFA via affecting stemness and the p53-dependent VEGFA/Dll4/Notch1 pathway to inhibit cancer cell angiogenesis.

## 1. Introduction

Liver cancer is one of the most malignant cancers in the world [1]. Millions of patients die from liver cancer because of lack of timely liver transplantation or hepatectomy [1]. In recent years, increasing clinical studies have confirmed that the 5-year overall survival rate of liver cancer less than 5 cm in diameter treated with radiofrequency ablation (RFA) is not inferior to that treated with hepatectomy; RFA also has a lower rate of complications and is noninvasive [2]. However, local recurrence and distant metastasis of residual cancer due to insufficient radiofrequency ablation (iRFA) remains an obstacle to overcome.

Residual cancer cells generally become more proliferative, motile, aggressive, and drug-resistant after iRFA [3]. These malignant changes in cells have been verified to confer stemness; that is, the fraction of liver cancer stem cells (LCSCs) substantially increased after iRFA [4]. Liu et al. have revealed that LCSCs induced by vascular endothelial growth factor A (VEGFA), which are produced under hypoxia and heat stimulation, accelerate the early recurrence of liver cancer after RFA treatment [5]. In addition, Kong et al. have reported that VEGFA secreted by altered liver cancer cells after heat treatment promotes the growth and angiogenesis of residual liver cancer after iRFA [6]. Thus, inhibiting VEGFA produced by LCSCs is a feasible and effective way to block the angiogenesis of residual liver cancer after iRFA treatment.

Angiogenesis is one of the crucial events for residual liver cancer development and growth [7]. VEGFA, as one of the most recognized and effective endothelial growth factors, mobilizes the endothelial progenitor cells to participate in tumor angiogenesis [7]. Uncontrollably, VEGFA secretion accelerates the malignant changes of cancer under hypoxia or heat stimulation [8]. Thus, VEGFA secretion is always through a complicated network involving in hypoxia-inducible factor 1, cytokines, hormones, etc. Recently, the regulatory effects of p53 on the expression of VEGFA have attracted increasing attention. Pal et al. have reported that p53 inhibits the expression of vascular permeability factor (VPF)/VEGF in mammary carcinoma by affecting their transcriptional activity under hypoxic conditions [9]. During sustained hypoxic conditions, Farhang et al. have reported that p53 reduces VEGFA production and inhibits angiogenesis through the p21/Rb pathway [10]. Thus, increasing the expression of p53 is a practical method to inhibit VEGFA-induced angiogenesis.

Amarogentin, a bioactive molecule extracted from *Swertia davidi Franch*, has been reported to activate p53 to promote apoptosis in liver cancer cells [11]. Our previous study has verified that amarogentin prevents the malignant transformation of liver cancer cells through upregulation of p53 [12]. In addition, amarogentin has been reported to prevent liver carcinogenesis via regulating LCSC renewal [13]. Furthermore, p53 suppresses tumor proliferation by inhibiting the expression of CD133 [14]. However, the roles and underlying mechanisms of amarogentin in inhibiting VEGFA-induced angiogenesis in residual liver cancer after iRFA treatment are poorly understood. Thus, in this study, we observed the percentage of LCSCs, the number of tubules produced by human umbilical vein endothelial cells (HUVECs), and the expression changes in the angiogenesis-related molecules VEGFA, delta-like ligand 4 (Dll4), and Notch1 in iRFA cancer cells and mouse models treated with amarogentin in both the presence and absence of p53 to reveal the antiangiogenesis effects and mechanisms of amarogentin in liver cancer after iRFA treatment.

## 2. Materials and Methods

### 2.1. Patients and Liver Cancer Samples

All 19 patients with liver cancer nodule (only 1 tumor nodule and less than 5 cm in diameter) were hospitalized at the Department of Hepatobiliary Surgery, the Third Hospital of Mianyang, from March 2016 to March 2018. Nine liver cancer patients were previously treated with RFA and later underwent hepatectomy after confirmation of iRFA by spiral computed tomography epigastric enhancement scanning. The remaining 10 liver cancer patients were only treated with hepatectomy. The use of patient liver cancer tissue samples in the present study was approved by the Ethics Committees of the Third Hospital of Mianyang. The clinical characteristics of patients with liver cancer are showed in Supplementary Table 1. There is no difference between the iRFA and hepatectomy groups.

### 2.2. Cell Culture

HepG2 and Huh7 cell lines were both purchased from the Cell Bank Type Culture Collection of the Chinese Academy of Sciences (Shanghai, China). Liver cancer cells were cultivated in a 37°C incubator at 5% CO_2_ and a suitable humidity level with DMEM containing 10% fetal bovine serum (FBS) (HyClone, USA).

### 2.3. iRFA Model and Amarogentin Treatment *In Vitro*

Sublethal heat treatment was used to mimic iRFA cells. That is, the iRFA cell model was generated as previously reported [8]. Briefly, HepG2 and Huh7 cells (5 × 10^4^) were plated in a 6-well plate and cultured for 12 h before incubation in a 50°C water bath for 10 min. Then, the cells were cultured at 37°C for 12, 24, and 48 h for subsequent experiments. For amarogentin (21018-84-8, PUSH Bio-Tec, China) treatment, the iRFA models of HepG2 and Huh7 cell lines were incubated in a 50°C water bath for 10 min before treatment with amarogentin (120 *μ*g/ml) for 24 h. The optimal effective dose of amarogentin for liver cancer cell lines was determined in our previous study [12].

### 2.4. Transfection Assay

A p53-shRNA plasmid containing the green fluorescence gene was purchased from GenePharma Inc. (Shanghai, China) and transfected with Lipofectamine®3000 (Thermo Fisher, USA) into Huh7 cells for 48 h. Then, iRFA cell model was generated as above. The transfection efficiency was greater than 70% (Supplementary Figure 1).

### 2.5. Tube Formation Assay

HUVECs were purchased from Procell Life Science & Technology Co. Ltd. (Wuhan, China). HUVECs (5 × 10^4^) were plated into a 48-well plate that was precoated with 50 *μ*l of Matrigel™ Basement Membrane Matrix (354234, BD Biosciences) and cultivated with a supernatant from the iRFA HepG2 and Huh7 cells for 6 h. The HUVECs tube formation was determined by optical microscopy and averaged from 5 fields. The quantification of tube formation was detected by ImageJ (NIH, USA).

### 2.6. Flow Cytometry Assay

The iRFA Huh7 cells (1 × 10^6^) were incubated with 10 *μ*l of phycoerythrin-CD133 antibody (372804, Biolegend, USA). The cells were incubated in a dark room at 4°C for 25 min before flow cytometry detection.

### 2.7. Enzyme-Linked Immunosorbent Assays

The supernatant levels of VEGFA (EK0539, Boster, China) were quantified using commercially available ELISA kits. For VEGFA detection in the supernatants, the concentrations were diluted to 20%. All the procedures were performed strictly in accordance with the manufacturer's instructions.

### 2.8. Animals

All BALB/c nu/nu male mice (8 weeks old, 22-25 g, specific pathogen-free) were purchased from the Experimental Animal Center of Chongqing Medical University. The mice were housed in sterile polycarbonate cages with free access to water and food with human care. All animal experimental procedures in the present study were approved by the Ethics Committee of Chongqing Medical University.

### 2.9. Xenograft and Amarogentin Treatment

A 1 × 10^7^ aliquot of normal Huh7 cells and iRFA model cells (48 h) (0.1 ml total volume) was injected subcutaneously into the left flank of each nude mouse. For amarogentin treatment, the mice were treated orally with amarogentin (0.2 *μ*g/g/d) as soon as they received the Huh7 cell xenograft. All mice were sacrificed after feeding for 30 days. Next, the tumors were removed for western blotting (WB) and immunohistochemical staining (IHC) assays. The optimal effective dose of amarogentin for mice with liver cancer was determined in previous studies [11–13].

### 2.10. Pathological and Immunohistochemical Analyses

Tumor tissues were fixed with 4% paraformaldehyde at 37°C for 48 h before being embedded in paraffin. The paraffin samples were cut into 3- to 5-*μ*m sections, followed by dewaxing and hydration. For histology, the sections were stained with hematoxylin and eosin (HE). For IHC, the sections were blocked with 5% BSA at 37°C for 2 h after denaturation of endogenous peroxidase was blocked with 30 ml/l hydrogen peroxide. Then, the sections were incubated with specific primary antibodies at 4°C overnight. Next, the sections were exposed to a horseradish peroxidase-conjugated secondary antibody, followed by incubation with a DAB detection kit (AR1026, Boster, China) at 37°C for 2-10 min. The details of the antibodies used are shown in Supplementary Table 2.

### 2.11. Western Blotting Analysis

Total protein was extracted with RIPA lysis buffer (AR0105; Boster, China) and separated on 10% sodium dodecyl-sulfate (SDS) polyacrylamide gels; then, the proteins were transferring onto polyvinylidene fluoride (PVDF) membranes and incubated with primary antibodies at 4°C overnight. Next, the PVDF membranes were reacted with horseradish peroxidase-conjugated anti-IgG secondary antibodies at 37°C for 2 h before incubation with enhanced chemiluminescence detection buffer (KGP1122; KEYGEN, China). The relative intensities of target protein bands were detected using a Chemico-EQ system (Bio-Rad, USA) and normalized to the amount of *β*-actin. The details of antibodies used are shown in Supplementary Table 2.

### 2.12. Reverse Transcriptase-Polymerase Chain Reaction Analysis

Total RNA was extracted using an ultrapure RNA kit (CW0597, Cwbiotech, China) and reverse transcribed into cDNA using a Primescript™ RT Reagent Kit with gDNA Eraser (RR047A, Takara, Japan). Polymerase chain reaction (PCR) was conducted using a SYBR Premix Ex Ta II Kit (RR820A, Takara, Japan) as follows: First, a total reaction system of 25 *μ*l was created by mixing 2×SYBR® Premix Ex Taq II (12.5 *μ*l), 10 *μ*mol/l forward primer(1 *μ*l), 10 *μ*mol/l reverse primer (1 *μ*l), cDNA (2 *μ*l), and RNase-free water (8.5 *μ*l); then, the mixture was denatured at 95°C for 30 s. Next, the mixture was subjected to 40 cycles of amplification at 95°C for 5 s and annealing at 60°C for 60 s. The relative expression levels of the target genes were determined using the 2(-Delta C(T)) method after normalization to the glyceraldehyde-phosphate dehydrogenase (GAPDH) gene. The primers of the target genes are shown in Supplementary Table 3.

### 2.13. Statistical Analysis

All data were expressed as the mean ± standard deviation (x ± s) and were analyzed using SPSS18.0 software (Chicago, Illinois, USA). Comparisons of multiple groups were performed with a single factor analysis of variance (one-way ANOVA), and pairs of independent samples were analyzed using Student's *t*-test. Differences were considered significant at a *p* value of less than 0.05.

## 3. Results

### 3.1. iRFA Promotes Angiogenesis via Inducing Stemness in Human Liver Cancer Tissues

**Figure 1:**
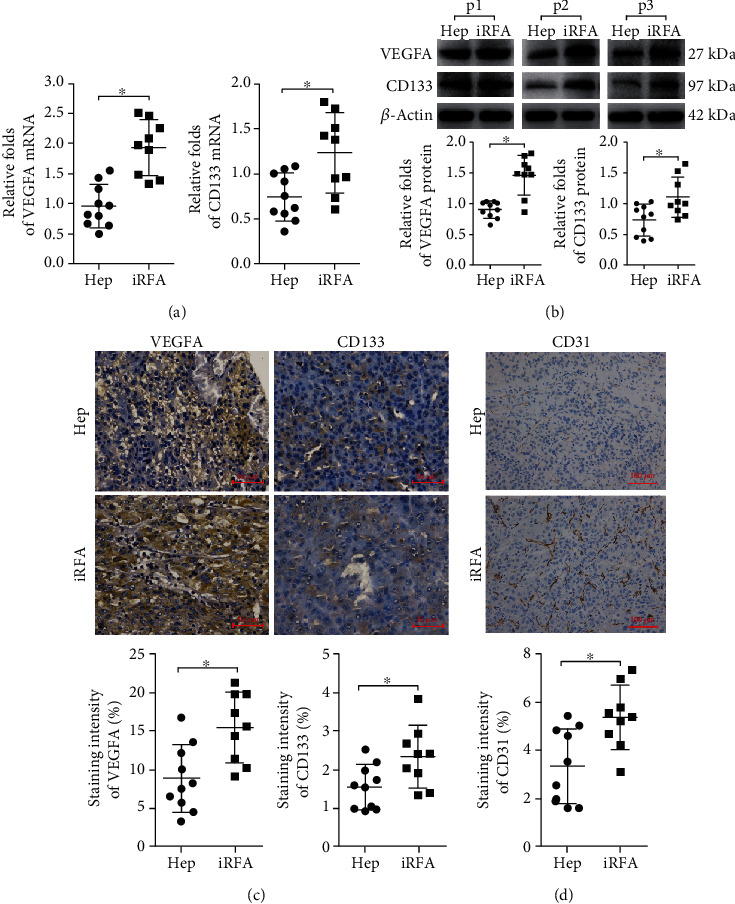
iRFA promotes angiogenesis via induction of stemness in human liver cancer tissues. (a) The mRNA expression levels of VEGFA and CD133 in iRFA liver cancer samples and hepatectomy liver cancer samples were detected by RT-PCR assay. (b, c) The protein expression levels of VEGFA and CD133 in the iRFA liver cancer samples and hepatectomy liver cancer samples were detected by WB and IHC (400x) assays. (d) The protein expression levels of CD31 in the iRFA liver cancer samples and hepatectomy liver cancer samples were detected by IHC (200x) assays (*p* < 0.05).

CD133 is a recognized surface marker for LCSCs [15]. The mRNA and protein expression levels of CD133 in the iRFA-liver cancer samples were higher than those in the hepatectomy-liver cancer sample (Figures 1(a)–1(c)). The mRNA and protein expression levels of VEGFA in the iRFA-liver cancer samples were higher than those in the hepatectomy-liver cancer samples (Figures 1(a)–1(c)). In addition, the protein levels of CD31 detected by IHC in the iRFA-liver cancer tissue were higher than those in the hepatectomy-liver cancer samples (Figure 1(d)). Thus, the expression trend for VEGFA and CD31 in the iRFA-liver cancer samples was similar to that for CD133, indicating that angiogenesis was promoted by LCSCs induced by iRFA.

### 3.2. Liver Cancer Stemness Induced by iRFA Facilitates Angiogenesis *In Vitro*

**Figure 2:**
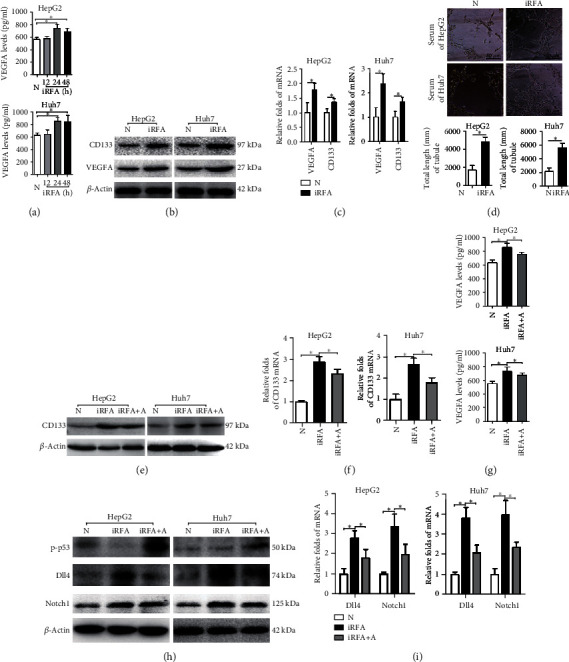
Amarogentin inhibits angiogenesis by decreasing the liver cancer cell stemness induced by iRFA. (a) The VEGFA supernatant levels in normal liver cancer cells and iRFA treatment cells at 12 h, 24 h, and 48 h were detected by ELISA. (b, c) The mRNA and protein expression levels of VEGFA and CD133 in normal liver cancer cells and iRFA cells at 48 h were detected by WB and reverse transcription-polymerase chain reaction (RT-PCR) assays. (d) HUVECs were cultured with supernatant from normal liver cancer cells and iRFA cells at 48 h to observe tube formation (400x). (e, f) The mRNA and protein expression levels of CD133 in normal liver cancer cells, iRFA cells, and iRFA cells treated with amarogentin were detected by WB and RT-PCR assays. (g) The VEGFA supernatant levels in normal liver cancer cells, iRFA cells, and iRFA cells treated with amarogentin were detected by ELISA. (h, i) The protein, phosphorylation, and mRNA expression levels of p53, Dll4, and Notch1 in normal liver cancer cells, iRFA cells (48 h), and iRFA cells treated with amarogentin were detected by WB and RT-PCR assays. N = normal liver cancer; iRFA = iRFA cells; iRFA+A = iRFA cells with amarogentin treatment group (*p* < 0.05).

In HepG2 cells, the supernatant levels of VEGFA after iRFA treatment at 24 h and 48 h were higher than those of nontreated cells (Figure 2(a)). Similarly, the mRNA and protein levels of CD133 and VEGFA in the iRFA-treated cells (24 h) were higher than those of nontreated cells (Figures 2(b) and 2(c)). In addition, the number of tubes formed by HUVECs cultured with the supernatant from iRFA-treated cells (24 h) was markedly higher than that formed by nontreated cells (Figure 2(d)). More dramatic changes in these indicators described above were also observed in Huh7 cells. Thus, the data indicated that the liver cancer cell stemness induced by iRFA treatment promoted angiogenesis *in vitro*.

### 3.3. Amarogentin Inhibits Angiogenesis by Decreasing the Liver Cancer Cell Stemness Induced by iRFA

The mRNA and protein levels of CD133 and the supernatant levels of VEGFA in iRFA-treated HepG2 cells were obviously decreased by amarogentin (Figure 2(e)). The same effects of amarogentin were observed in iRFA-treated Huh7 cells. In addition, the mRNA and protein levels of Dll4 and Notch1 in iRFA-treated cells were decreased by amarogentin, and phosphorylated p53 levels were increased (Figure 2(f)). Thus, the data suggested that amarogentin inhibited angiogenesis by decreasing the liver cancer cell stemness induced by iRFA.

### 3.4. Amarogentin Suppresses Liver Cancer Growth by Inhibiting Angiogenesis in Xenograft Mice

**Figure 3:**
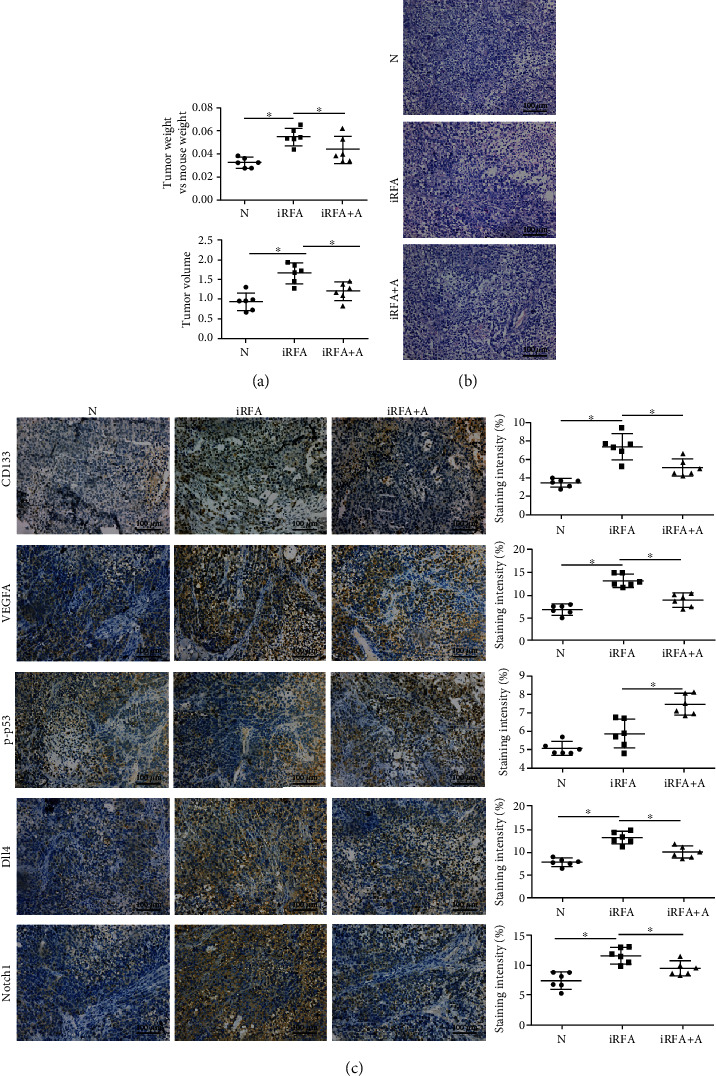
Amarogentin suppresses liver cancer growth by inhibiting angiogenesis in xenograft mice. (a) The tumor weights and volume were significantly higher in iRFA model mice than in mice treated with amarogentin. (b) HE staining was used to observe the tumor tissues. (c) The protein and phosphorylation levels of CD133, VEGFA, p53, Dll4, and Notch1 were detected by IHC assay in the normal Huh7 cell xenograft group, the iRFA cell xenograft group, and the iRFA cell xenograft treated with amarogentin group (400x). N = normal Huh7 cell xenograft group; iRFA = iRFA cell xenograft group; iRFA+A = iRFA cell xenograft treated with amarogentin group (*p* < 0.05).

The tumor weights and volumes of the iRFA model mice were significantly decreased by amarogentin (Figure 3(a) and Supplementary Figure 2). Consistently, the expression levels of CD133, VEGFA, Dll4, and Notch1 in iRFA tumor tissues were decreased by amarogentin, and phosphorylated p53 levels were increased (Figures 3(b) and 3(c)). Thus, these data implied that amarogentin suppresses liver cancer growth by inhibiting angiogenesis in xenograft mice.

### 3.5. Amarogentin Inhibits iRFA-Induced Angiogenesis via Affecting the p53-Dependent VEGFA/Dll4/Notch1 Pathway

**Figure 4:**
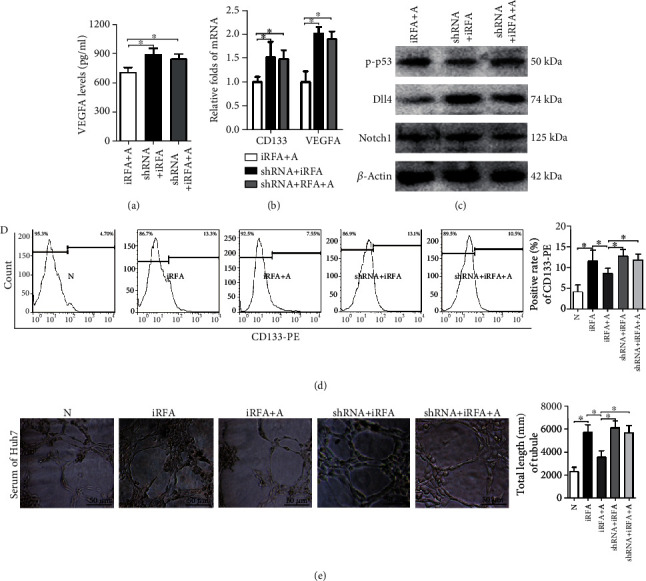
Amarogentin inhibits iRFA-induced angiogenesis via the p53-dependent VEGFA/Dll4/Notch1 pathway in Huh7 cells. (a–c) The supernatant levels of VEGFA, the mRNA levels of CD133 and VEGFA, and the protein and phosphorylation levels of p53, Dll4, and Notch1 in iRFA cells treated with amarogentin, iRFA cells transfected with p53-shRNA, and iRFA cells transfected with p53-shRNA before treatment with amarogentin were detected by ELISA, RT-PCR, and WB assays, respectively. (d) The percentages of LCSCs in normal liver cancer cells, iRFA cells, iRFA cells treated with amarogentin, iRFA cells transfected with p53-shRNA, and iRFA cells transfected with p53-shRNA before treatment with amarogentin were indicated by CD133-PE staining and detected by flow cytometry. (e) HUVECs were cultured with supernatant from normal liver cancer cells, iRFA cells, iRFA cells treated with amarogentin, iRFA cells transfected with p53-shRNA, and iRFA cells transfected with p53-shRNA before treatment with amarogentin (400x). N = normal liver cancer; iRFA = iRFA cells; iRFA+A = iRFA cells with amarogentin treatment group; iRFA+shRNA = iRFA cells iRFA cells transfected with p53-shRNA group; iRFA+shRNA+A = iRFA cells transfected with p53-shRNA before treatment with amarogentin group (*p* < 0.05).

The effects of amarogentin on the fraction of CD133-positive cells and the mRNA levels of VEGFA, CD133, Dll4, and Notch1 after iRFA treatment were also downregulated by p53 knockdown in Huh7 cells (Figures 4(a)–4(d)). Similarly, the effects of amarogentin on the number of tubes formed by HUVECs cultured with supernatant from iRFA model cells were counteracted by p53 knockdown in iRFA models of Huh7 cells (Figure 4(e)). Thus, these data indicated that amarogentin inhibited iRFA-induced angiogenesis via affecting the p53-dependent VEGFA/Dll4/Notch1 pathway.

## 4. Discussion

Although RFA is the most effective method for treating liver cancer other than liver transplantation and hepatectomy, an effective solution has not been found for the occurrence of residual cancer. The heat produced by RFA is not enough to kill marginal cancer cells; instead, it increases the malignant progression of liver cancer [3]. Sublethal heat treatment promotes liver cancer cell development of a spindle-like morphology and transformation CD133-positive liver cancer cells, which are progenitor-like cancer cells that are highly proliferative [4]. Tong et al. have asserted that residual liver cancer cells show a higher percentage of stem-like cells and have levels of invasiveness, metastasis, and drug resistance [15]. In addition, Wang et al. have reported that downregulating the expression of CD133 suppressed proliferation, invasion, and autophagy in iRFA-treated liver cancer [4]. Consistently, in our study, we have observed that the mRNA and protein levels of CD133 were higher in the iRFA liver cancer samples. Moreover, we have observed that the fraction of CD133-positive cells was markedly increased by iRFA treatment. Thus, the cancer cells treated with iRFA generally achieve stemness. That is, more liver cancer cells transform into LCSCs.

Cancer stem cells (CSCs), or tumor-initiating cells (TICs), are regarded as the origin of cancer and are thought to cause treatment tolerance [16]. In addition to the stronger proliferation, invasion, metastasis, differentiation, and drug resistance of CSCs, promotion of tumor angiogenesis is another main feature. Eyler et al. and Atala et al. have found that breast CSC-derived endothelial cells contribute to tumor angiogenesis [17, 18]. Zheng et al. have reported that downregulation of CD13, a marker for LCSCs, inhibits the growth of liver cancer by killing LCSCs and suppressing angiogenesis [7]. In addition, Kong et al. have confirmed that tumor-associated endothelial cells enhance angiogenesis and promote the invasiveness of residual liver cancer after iRFA treatment [19]. In the present study, we have observed that residual liver cancer angiogenesis is promoted by LCSCs induced by iRFA. Moreover, the increased fraction of LCSCs facilitates angiogenesis in iRFA-treated cells, further promoting liver cancer growth in xenograft mice. Thus, killing CSCs is an unquestionably effective method to retard the angiogenesis of residual liver cancer, as well as the recurrence and metastasis of residual liver cancer.

Amarogentin, an anticancer compound extracted from *Swertia davidi Franch*, has been reported to inhibit liver cancer, cervical cancer, gastric carcinoma, and skin carcinogenesis *in vivo* and *in vitro* [11, 12, 20–22]. Sur et al. have confirmed that amarogentin significantly reduces the numbers of LCSCs in both the pre- and postinitiation stages of carcinogenesis [13]. Park et al. have reported that the antiproliferative effects of p53 are antagonized by rescuing CD133 expression in a p53-overexpressing cell line [14]. Their results have indicated that the tumor-suppressive activity of p53 might be mediated by CD133 suppression. p53, as a most important tumor-suppressing gene, inhibits tumorigenesis by activating a lot of effector pathways. Although mutant p53 is known to be transcriptionally inactive and promotes the initiation and progression of cancer, phosphorylated p53 as a transcriptional factor plays an important role in tumor inhibition. Importantly, our previous study has verified that amarogentin prevents the malignant transformation of liver cancer cells through upregulating p53. Moreover, we have observed that the number of CD133-positive cells is obviously decreased by amarogentin in both HepG2 and Huh7 cells, accompanied by increased p53 phosphorylation and angiogenesis inhibition. However, the mechanism that is involved in amarogentin inhibition of liver cancer angiogenesis upon iRFA treatment via killing LCSCs requires further clarification.

As a highly specific vascular endothelial growth factor, VEGFA is involved in various conditions of angiogenesis via initiating the Dll4/Notch1 pathway [23]. Inflammation and hypoxia are characteristics of the tumor microenvironment, but they also promote VEGFA secretion in cancer cells [24]. Liu et al. have reported that VEGFA induced by iRFA promotes tumor stemness and accelerates tumorigenesis in liver cancer cells [5]. In addition, Kong et al. have reported that the hypoxic-inducible factor 1*α* (HIF1*α*)/VEGFA pathway was involved in the angiogenesis of residual liver cancer after iRFA treatment, and bevacizumab, which targets VEGFA, inhibited tumor growth and angiogenesis in iRFA model cells [6]. Thus, VEGFA should be an appropriate target for antitumor therapy. Pfaff et al. have reported that augmentation of p53 expression could decrease the levels of VEGFA in an ischemia-induced angiogenesis and arteriogenesis mouse model [25]. However, the roles of p53 in the regulation of VEGFA have always been controversial. Quite a few studies have reported that p53 inhibits the expression of VEGFA in several solid tumors [9, 10]. Other studies have reported that the expression of p53 is positively related to the expression of VEGFA in lung cancer and renal carcinoma [26, 27]. These controversial results may be due to the lack of differentiation between mutations and nonmutations in p53. In our study, we have founded that the phosphorylation levels of p53 oppose the levels of VEGFA in iRFA model cells. After amarogentin treatment, the expression of p53 was increased, leading to decreases in CD133 and VEGFA. In addition, the effects of amarogentin on the inhibition of VEGFA were counteracted by silencing p53 in CD133-positive cells after iRFA treatment. In addition, the expression levels of the angiogenesis-related molecules Dll4 and Notch1 were homodromous to the expression of VEGFA. Thus, amarogentin suppresses liver cancer growth after iRFA treatment by affecting the p53-dependent VEGFA/Dll4/Notch1 pathway to inhibit tumor angiogenesis. This may be the mechanism by which amarogentin inhibits liver cancer angiogenesis after iRFA treatment by killing LCSCs.

In conclusion, our present results suggest that amarogentin affects liver cancer angiogenesis via the p53-dependent VEGFA/Dll4/Notch1 pathway in CD133-positive cells after iRFA treatment, implying a novel supplementary strategy for the treatment of residual liver cancer after iRFA treatment.

## Data Availability

The datasets used and/or analyzed during the current study are available from the corresponding author on reasonable request.

## Supplementary Materials

Supplementary Materials Supplementary Figure 1 A and B: transfection efficiency was detected by fluorescence microscopy and western blotting assay (WB) after vector and p53-shRNA transfection for 48 h. Supplementary Figure 2 A: subcutaneous tumors after sacrificing the xenograft mice at 30 days. Supplementary Figure 2 B: three mice were recorded after xenograft at 3 weeks. Supplementary Table 1: clinical characteristics of patients with liver cancer. Supplementary Table 2: antibodies and dilution ratio. Supplementary Table 3: primers for targeted genes.
