# Supplementary material for: Amarogentin Inhibits Liver Cancer Cell Angiogenesis after Insufficient Radiofrequency Ablation via Affecting Stemness and the p53-Dependent VEGFA/Dll4/Notch1 Pathway
Source: Biomed Res Int. 2020 Oct 20;2020:5391058. doi: 10.1155/2020/5391058 (PMC7596460; doi:10.1155/2020/5391058)
Supplement: Supplementary Materials — Supplementary Figure 1 A and B: transfection efficiency was detected by fluorescence microscopy and western blotting assay (WB) after vector and p53-shRNA transfection for 48 h. Supplementary Figure 2 A: subcutaneous tumors after sacrificing the xenograft mice at 30 days. Supplementary Figure 2 B: three mice were recorded after xenograft at 3 weeks. Supplementary Table 1: clinical characteristics of patients with liver cancer. Supplementary Table 2: antibodies and dilution ratio. Supplementary Table 3: primers for targeted genes. [file 5391058.f1.pdf]

Amarogentin inhibits liver cancer cell angiogenesis after insufficient radiofrequency ablation via affecting stemness and the p53-dependent VEGFA/Dll4/Notch1 pathway

Jin Wang[1], Yinglin Zhang[2], Yongchuan Zhang[2,*], Haitao Gu[3,*]

Supplementary materials

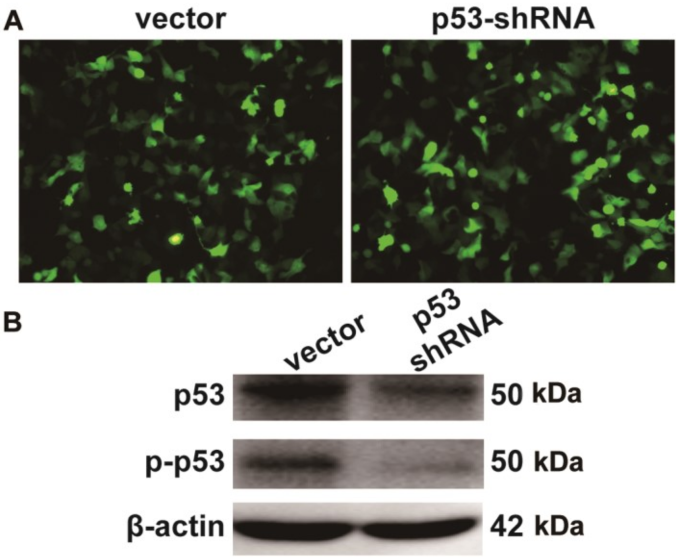

Supplementary figure 1. A and B, Transfection efficiency was detected by fluorescence microscopy and western blotting assay (WB) after vector and p53-shRNA transfection for 48 h.

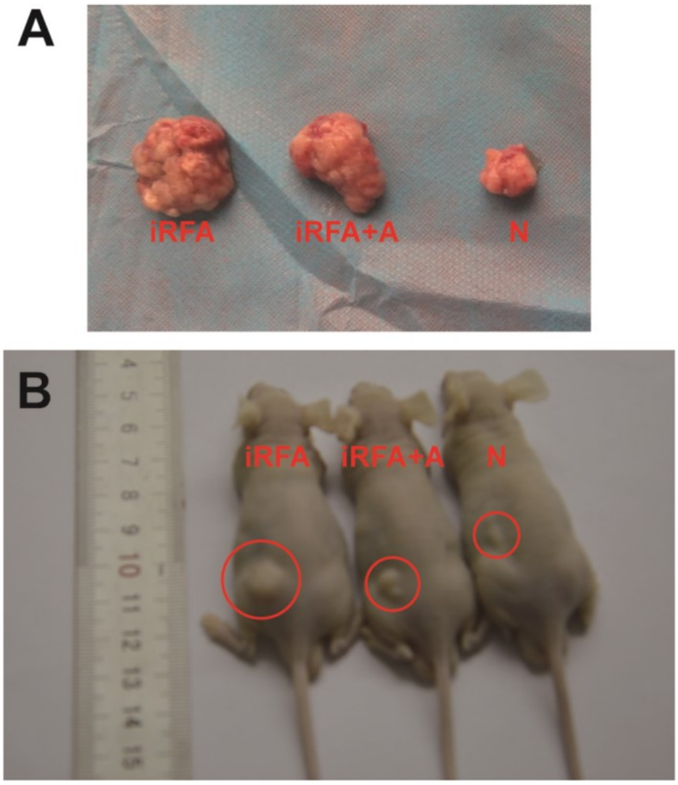

Supplementary figure 2. A, Subcutaneous tumors after sacrificing the xenograft mice at 30 days. B, three mice were recorded after xenograft at 3 weeks.

Supplementary table 1. Clinical characteristics of patients with liver cancer

characteristics	iRFA	HEP	χ^2	P
Number	9	10	-	-
Gender (male/ female)	5/4	6/4	-	-
Number of cirrhosis	3	4	-	-
Child-Pugh score (A/B)	8/1	9/1	-	-
Age (years)	48.28±6.52	45.74±5.18	1.625	0.097
Diameter of tumor	2.21±0.83	2.05±0.54	2.033	0.085
HBV DNA (log_{10} copies/ml)	2.73±0.19	4.15±0.46	2.569	0.291
IGGR-15 (%)	10.63±7.84	12.44±8.61	0.715	0.362
AFP (ng/ml)	276.39±54.57	312.60±37.19	1.312	0.513
ALT (IU/L)	32.64±11.83	40.35±13.67	2.164	0.196
Albumin (g/L)	38.73±6.16	40.37±8.90	0.673	0.512
TBIL (umol/L)	15.14±4.82	17.48±6.43	2.316	0.347

Supplementary table 2. Antibodies and dilution ratio

Antibodies	Application
Rabbit anti-VEGFA antibody (ab52917, Abcam, USA)	1:8000 for WB; 1:100 for IHC
Rabbit anti-CD133 antibody (ab216323, Abcam, USA)	1:1000 for WB; 1:500 for IHC
Rabbit anti-CD31 antibody (ab134168, Abcam, USA)	1:500 for IHC
PE anti-CD133 Antibody (372804, Biolegend, USA)	1:20 for FCM
Rabbit anti-p-p53 antibody (ab76242, Abcam, USA)	1:1000 for WB; 1:100 for IHC
Rabbit anti-p53 antibody (ab32389, Abcam, USA)	1:1000 for WB
Rabbit anti-Dll4 antibody (ab176876, Abcam, USA)	1:500 for WB; 1:100 for IHC
Rabbit anti-p-AKT antibody (ab52627, Abcam, USA)	1:1000 for WB; 1:100 for IHC
Rabbit anti-β-actin antibody (BM0627, Boster, China)	1:200 for WB
Goat anti-rabbit IgG HRP antibody (BA1055, Boster, China)	1:2000 for WB

Supplementary table 3. Primers for targeted genes

Targeted genes	Primers	Length (bp)
CD133	Forward: 5′-AGTCGGAAACTGGCAGATAGC-3′	99
	Reverse: 5′-GGTAGTGTTGTACTGGGCCAAT-3′	
VEGFA	Forward: 5′-AGGGCAGAATCATCACGAAGT-3′	75
	Reverse: 5′-AGGGTCTCGATTGGATGGCA-3′	
Dll4	Forward: 5′-GTCTCCACGCCGGTATTGG-3′	98
	Reverse: 5′-CAGGTGAAATTGAAGGGCAGT-3′	
Notch1	Forward: 5′-GAGGCGTGGCAGACTATGC-3′	140
	Reverse: 5′-CTTGTACTCCGTCAGCGTGA-3′	
GAPDH	Forward: 5′-TGTGGGCATCAATGGATTTGG-3′	116
	Reverse: 5′-ACACCATGTATTCCGGGTCAAT -3′	
